# Fire Service Instructor's undergarment choice can minimise physiological and perceptual strain

**DOI:** 10.1186/2046-7648-4-S1-A67

**Published:** 2015-09-14

**Authors:** Emily Watkins, Alan Richardson

**Affiliations:** 1 Environmental Extremes Laboratory, Centre for Sport and Exercise Science and Medicine, University of Brighton, Eastbourne, UK

## Introduction

The South East Regional Fire Service requested an investigation into the effect of different undergarments worn by fire service instructors, to help improve thermoregulation and reduce the strain experienced. Literature suggests that wearing shorts and t-shirt may reduce heat strain [1], whilst no research has yet established the effect of wearing compression undergarments in fire environments. The study aimed to identify which type of undergarment [boiler suit (BOILER), whole body compression garments (COMPRESSION) or shorts and t-shirt (SHORTS)] produced the least physiological and perceptual strain.

## Methods

Eight males (age 20 ± 2 years; weight 75.7 ± 7.1 kg; height 177 ± 7 cm) were monitored during three 45 mins sessions in a heat chamber (49.5 ± 1.4 °C and 16.9 ± 4.3 % rh) whilst performing intermittent exercise [5 mins walking (4 km.h¯¹, 1 % gradient) and 5 mins rest]. Participants wore fire service kit and a rucksack to replicate a breathing apparatus, weighing 17 kg in total. Physiological and perceptual measures were recorded every 5 min. Venous blood samples were collected before and after heat exposure for analysis of interleukin (IL)-6.

## Results

Two way repeated measures ANOVA's were conducted, and revealed significant interactions for change in heart rate, change in rectal temperature (ΔT_re_), volume of oxygen uptake (VO_2_), physiological strain index (PSI) and IL-6, p < 0.05. IL-6 was significantly decreased for COMPRESSION (6.45 ± 0.43 pg.mL¯¹) and SHORTS (6.59 ± 0.30 pg.mL¯¹) compared to BOILER (6.96 ± 0.28 pg.mL¯¹), p < 0.05. Significant differences were also present between garment types at 45min for PSI and ΔT_re_, with trends suggesting COMPRESSION caused the lowest levels of strain (4.06 ± 0.85 °C, and 0.70 ± 0.31 °C, respectively) compared to SHORTS (4.50 ± 1.07 °C and 0.76 ± 0.37 °C, respectively) and BOILER (5.07 ± 1.02 °C and 1.00 ± 0.56 °C, respectively), p < 0.05. Thermal sensation (TSS) trends suggest that COMPRESSION (7.13 ± 0.52) generated less perceptual stress in comparison to SHORTS (7.43 ± 0.45) and BOILER (7.75 ± 0.27), p > 0.05.

## Discussion

Previous studies have noted no thermoregulatory improvement whilst wearing COMPRESSION in sporting situations[2,3]. However, this study suggests that in hot environments, with protective clothing, wearing COMPRESSION may be beneficial, possibly due to the thin material, tight fit, and wicking capabilities of the fabric.

## Conclusion

In comparison to standard issue boiler suits or shorts and t-shirt, wearing compression garments underneath protective clothing, during fire-fighting operations, significantly improves thermoregulation, reducing physiological strain and inflammation. Undergarment selection has a less pronounced effect on perceptions of stress; however differences may be meaningful to fire service instructors.
